# Neutropenia induced in outbred mice by a simplified low-dose cyclophosphamide regimen: characterization and applicability to diverse experimental models of infectious diseases

**DOI:** 10.1186/1471-2334-6-55

**Published:** 2006-03-17

**Authors:** Andres F Zuluaga, Beatriz E Salazar, Carlos A Rodriguez, Ana X Zapata, Maria Agudelo, Omar Vesga

**Affiliations:** 1 GRIPE: Infectious Disease Problems Research Group, University of Antioquia Medical School, Medellín, Colombia; 2 Department of Pharmacology & Toxicology, University of Antioquia Medical School, Medellín, Colombia; 3 Section of Infectious Diseases, Department of Internal Medicine, University of Antioquia Medical School, Medellín, Colombia

## Abstract

**Background:**

For its low cost and ease of handling, the mouse remains the preferred experimental animal for preclinical tests. To avoid the interaction of the animal immune system, in vivo antibiotic pharmacodynamic studies often employ cyclophosphamide (CPM) to induce neutropenia. Although high doses (350–450 mg/kg) are still used and their effects on mouse leukocytes have been described, a lower dose (250 mg/kg) is widely preferred today, but the characteristics and applicability of this approach in outbred mice have not been determined.

**Methods:**

Fifteen female ICR mice were injected intraperitoneally with 150 and 100 mg/kg of CPM on days 1 and 4, respectively. Blood samples (~160 μL) were drawn from the retro-orbital sinus of each mouse on days 1, 4, 5, 6, 7 and 11. Leukocytes were counted manually and the number of granulocytes was based on microscopic examination of Wright-stained smears. The impact of neutropenia induced by this method was then determined with a variety of pathogens in three different murine models of human infections: pneumonia (*Klebsiella pneumoniae*, *Streptococcus pneumoniae*, *Staphylococcus aureus*), meningoencephalitis (*S. pneumoniae*), and the thigh model (*S. aureus*, *Escherichia coli*, *Bacteroides fragilis*).

**Results:**

The basal count of leukocytes was within the normal range for outbred mice. On day 4, there was an 84% reduction in total white blood cells, and by day 5 the leukopenia reached its nadir (370 ± 84 cells/mm^3^). Profound neutropenia (≤10 neutrophils/mm^3^) was demonstrated at day 4 and persisted through days 5 and 6. Lymphocytes and monocytes had a 92% and 96% decline between days 1 and 5, respectively. Leukocytes recovered completely by day 11. Mice immunosupressed under this protocol displayed clinical and microbiological patterns of progressive and lethal infectious diseases after inoculation in different organs with diverse human pathogens.

**Conclusion:**

A CPM total dose of 250 mg/kg is sufficient to induce profound and sustained neutropenia (<10 neutrophils/mm^3^) at least during 3 days in outbred mice, is simpler than previously described methods, and allows successful induction of infection in a variety of experimental models.

## Background

Currently, animal models of infection are considered essential for the study of physiopathology and pharmacology of infectious disease, being the best tool to bridge in vitro studies and clinical trials [1]. The neutropenic mouse thigh infection model has been extensively used, and its impact on the current knowledge of antimicrobial pharmacology is undisputable [2]. It allowed the quantitative determination of pharmacokinetic (PK) and pharmacodynamic (PD) indices (PK/PD) useful to predict antibiotic efficacy while minimizing selection of resistant mutants [2]. This revolutionary advance in pharmacology was reached after characterizing the complex relationship between host, drug and pathogen, thanks to the unique opportunity offered by animal models for comparing the responses to infection and therapy under several immune statuses [3].

Antibiotic activity can be positively influenced by the host immune system. For instance, the magnitude of the PK/PD index necessary for successful therapy is reduced in animal models by the presence of neutrophils [3,4]. Thus, in order to study in vivo the intrinsic activity of antibiotics without the influence of the immune system, animals are commonly rendered neutropenic with cytotoxic agents like 5-fluorouracil, methotrexate, and cyclophosphamide (CPM). In fact, the latter is the most frequently used immunosuppressive agent in experimental anti-infective pharmacology. CPM is a prodrug requiring metabolic transformation to generate active alkylating species. The initial activation is mediated by hepatic cytochrome P-450 enzymes and is the major pathway of elimination of the parent drug. Hydroxylation at the carbon-4 position of the oxazaphosphorine ring produces 4-hydroxycyclophosphamide in equilibrium with the tautomer aldophosphamide, which spontaneously degrades to phosphoramide mustard and acrolein [5]. These alkylating species bind to DNA and induce strand breakage and cross-linking, killing actively replicating cells [6].

Although the effects in mice of high doses of CPM (350–450 mg/kg) have already been described [7] and some authors still use them [8-10], a lower dose (250 mg/kg) is preferred nowadays [11-13]. The low dose regimen is derived from a rat model [14,15], but the length and extent of the induced neutropenia in outbred mice have not been characterized in detail [16]. At the same time, outbred mice are preferred for infection models because their genetic diversity allows determination of individual responses to infection and treatment. Such individuality constitutes another reason to study their response to the method of immunosupression.

The aim of this study was to evaluate the efficacy of a simplified 250 mg/kg regimen of CPM for murine immunosupression, characterizing its effects on white blood cells, including neutrophils, lymphocytes and monocytes, and its impact in three different animal models of infection with a variety of human pathogens.

## Methods

### Mice

For all experiments we used 6 week-old, female, murine pathogen free Swiss albino mice, belonging to the strain Udea:ICR(CD-1), and weighting 23–27 g [17]. All mice received sterile food and water ad libitum and were housed individually during the experiments. The study was approved by the University of Antioquia Animal Care and Experimentation Committee.

### Cyclophosphamide treatment

CPM powder (Cytoxan^®^, Bristol Myers Squibb, NY, USA) was dissolved in distilled USP water for injection to a final concentration of 20 mg/mL. Fifteen animals were subjected to CPM treatment in order to have 10 mice for daily bleeding and 5 reserved to replace any dead animal. All mice received a total dose of 250 mg/kg by two 0.5 mL intraperitoneal injections scheduled at day 1 (150 mg/kg) and day 4 (100 mg/kg).

### Blood leukocytes counts

Blood samples (~160 μL) were taken from the retro-orbital plexus in heparinized capillary tubes (Modulohm A/S, Herlev, Denmark) at 9:00 AM on days 1, 4, 5, 6, 7 and 11. Total and differential white blood cell counts (neutrophils, lymphocytes and monocytes) were performed manually for each sample using a Neubauer chamber (Brand GMBH, Wertheim, Germany) and microscopic examination of Wright-stained smears with 100X objective [18].

### Animal models of infection

#### Neutropenic mouse thigh infection model

we tested the applicability of this immunosupression method inducing sepsis with three different pathogens and using the number of microorganisms growing in the thighs as the end-point. For the thigh model, all mice were infected on day 5 of immunosupression, 16 hours after the second dose of CPM.

First, to determine bacterial growth along time in the tissues of neutropenic mice with and without antibiotic treatment, we designed an in vivo time-kill curve experiment with this model. The thighs of 36 anaesthetized mice were inoculated with a 100 μL suspension containing 10^4–5 ^colony forming units per milliliter (CFU/mL) of log-phased *Staphylococcus aureus *GRP-0057, a wild-type clinical isolate. Four untreated mice were sacrificed at the time of infection and when treatment started two hours later. The remaining 32 animals were randomized (16 mice each group) to treatment with sterile saline or vancomycin (Vancocin^®^CP, Ely Lilly & Co., Mexico, D.F.), 1200 mg/kg/day administered by subcutaneous injections divided every 3 hours. Two animals from each group were sacrificed at 3, 6, 9, 12, 15, 18, 21, and 24 hours after starting treatment, and their thighs dissected, homogenized, and properly diluted for agar plating, incubation, and colony counting (CFU/g).

Second, we determined the size of the inoculum needed to successfully induce disease with *E. coli *SIG-1, a clinical isolate resistant to ampicillin, ampicillin-sulbactam, and first-generation cephalosporins. Groups of 6 neutropenic mice were inoculated in the thighs with 2, 3, 4, 5, and 6 logs of bacteria, and 2 animals from each inoculum group were killed immediately after infection (hour 0) and 2 and 26 hours later. The experiment for the group inoculated with 4 logs was repeated in a different time with another group of 6 mice to test for reproducibility. The thighs were harvested as described above.

Third, we infected the mice with *B. fragilis *ATCC 25285 (8.0 log_10 _CFU/mL) mixed with *E. coli *SIG-1 (6.0 log_10 _CFU/mL) to measure the extent to which bacterial synergism could be achieved in neutropenic animals. Of note, anaerobes do not grow in the thighs of neutropenic outbred mice without a second, usually aerobe pathogen. Sixteen neutropenic mice were inoculated in the thighs to determine the number of viable *B. fragilis *cells right after infection (hour 0) and at 2, 4, 6, 12, 18, 23, and 26 hours (2 mice per time-point). Except for the anaerobic techniques required to handle and culture *B. fragilis*, thigh harvesting followed the routine described above.

#### Pneumonia models

we inoculated neutropenic mice with aerosolized *Klebsiella pneumoniae *GRP-0107 (a wild-type clinical isolate) and with nasal instillation of *Streptococcus pneumoniae *INS-E611 (a penicillin-resistant strain obtained from a patient with meningitis) or *Staphylococcus aureus *ATCC 29213. For the first model, we exposed 10 mice during 45 minutes to bacterial aerosols produced by a Collison nebulizer from a log-phased suspension with ~10^9 ^CFU/mL, starting 24 hours after the second dose of CPM (day 5). Two animals were sacrificed randomly at the time of infection and 14, 28, 34 and 38 hours later. For the second model, ten neutropenic and deeply anesthetized mice were instilled 50 μL log-phased bacterial suspension by the nasal route 16 hours after the second dose of CPM (day 5). Two mice were sacrificed at the time of infection and 6, 14, 24 and 38 hours later. The lungs were harvested and properly diluted and plated, in a similar manner as described for the thigh model.

#### Meningoencephalitis model

to determine the impact of severe neutropenia induced by this protocol on the reproducibility of an experimental infection, we modified a model of acute meningoencephalitis [19] and compared bacterial growth in mice with severe neutropenia (CPM group) and normal leukocyte counts (control group). We chose this model because the method for bacterial inoculation allows induction of successful infection even in normal mice. Animals from CPM and control groups (52 and 45 animals, respectively) were inoculated directly into the frontal lobe by supra-retro orbital injection of 50 μL bacterial suspension containing 10^5–6 ^CFU/mL of *S. pneumoniae *GRP-0056, a trimethoprim-sulfamethoxazole resistant clinical isolate. At least two animals were killed at the time of infection and every 1 hour during five hours afterwards (CPM group), and then every 6 hours (both groups) during 36 hours. Brains were harvested and plated in a similar fashion as described for the thigh model, accounting for proper dilutions to express data as CFU/g.

## Results

### General aspects

Ten mice were bled as scheduled up to days 5 and 6, when 2 and 3 animals respectively had to be replaced. The reason to replace these 5 animals was clinical deterioration due to blood loss. On day 11, one animal had systemic signs of infection and was excluded from the last bleeding point.

### Verification of leukopenia

The basal count of leukocytes was determined at day 1, 8 hours before the first dose of CPM (150 mg/kg). Total and differential white blood cells counts were within the normal range for outbred mice [20]. On day 4, 8 hours before the second injection of CPM (100 mg/kg), there was an 84% reduction in total white blood cells. Leukopenia reached the nadir at day 5 (370 ± 84/mm^3^), 16 hours after the second dose of CPM. This is particularly important for the thigh infection model, because the procedure for bacterial inoculation takes place on the fifth day at this time (9:00 AM). Leukocytes began to recover at day 6 and reached normal values by day 11 (Table 1).

**Table 1:** Leukocytes counts of female Udea:ICR(CD-1) mice at baseline and after treatment with a low-dose cyclophosphamide regimen. Total number of leukocytes (cells/mm^3^) and differential count in peripheral blood of 10 mice at baseline in day 1 (8 hours prior to first dose), at day 4 (8 hours prior to second dose), during immunosupression (days 5–7), and at recovery (day 11), compared with reference values for 6 week-old mice.

**White blood-cell Population**	**Cells/mm^3 ^(± SEM)**						**Normal Range**
		
	**Day 1**	**Day 4**	**Day 5**	**Day 6**	**Day 7**	**Day 11**	
**Total Leukocytes**	5155 (405)	845 (104)	370 (84)	610 (94)	620 (179)	5863 (1103)	1000–5000
**Neutrophils**	528 (50)	0 (0)	10 (10)	0 (0)	36 (31)	1700 (321)	110–2150
**Lymphocytes**	4256 (395)	831 (100)	337 (72)	517 (95)	419 (124)	2905 (415)	500–4300
**Monocytes**	371 (74)	14 (7)	14 (9)	93 (29)	166 (76)	1248 (604)	10–300

### Neutrophils, lymphocytes and monocytes

**Figure 1:**
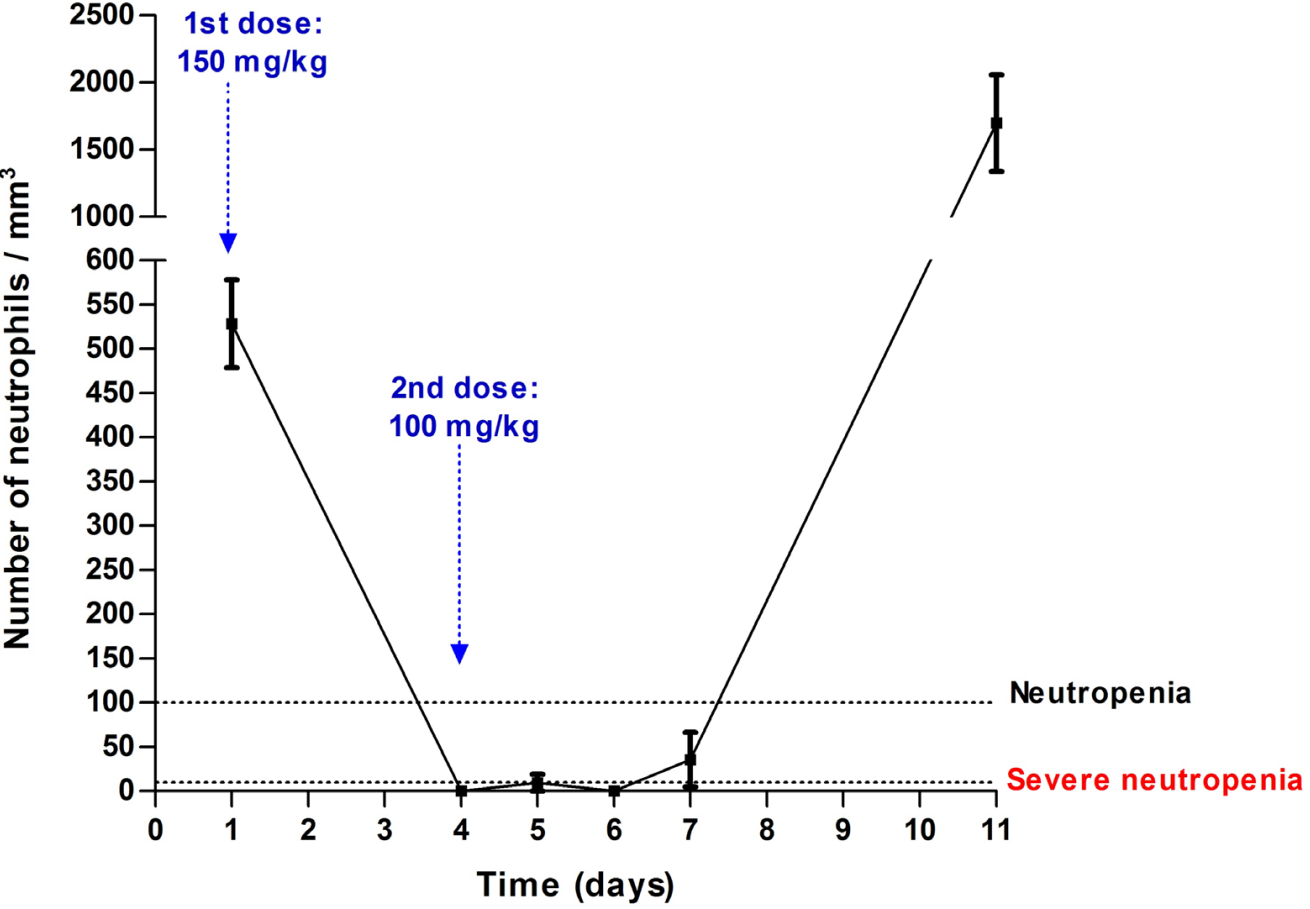
Length and extent of neutropenia induced in female ICR mice after a low-dose cyclophosphamide regimen. Total number of neutrophils in peripheral blood of 10 mice along 11 days after 2 intraperitoneal injections of cyclophosphamide. Data-points represent means ± standard error of the mean.

The initial number of neutrophils in normal mice was 528 ± 50 cells/mm^3^. Figure 1 illustrates how CPM 250 mg/kg induced severe neutropenia (≤10 neutrophils/mm^3^) by day 4 and the absolute neutrophils count remained below this value throughout days 5 and 6 for all animals. By day 7, mice had 36 ± 31 neutrophils/mm^3^, and at day 11 they all had returned gradually to normal but greater than baseline values (1700 ± 321 cells/mm^3^). Lymphocyte and monocyte counts declined 92% and 96% by day 1 and 5, respectively, and started to recover after day 7.

### Animal models of infection

**Figure 2:**
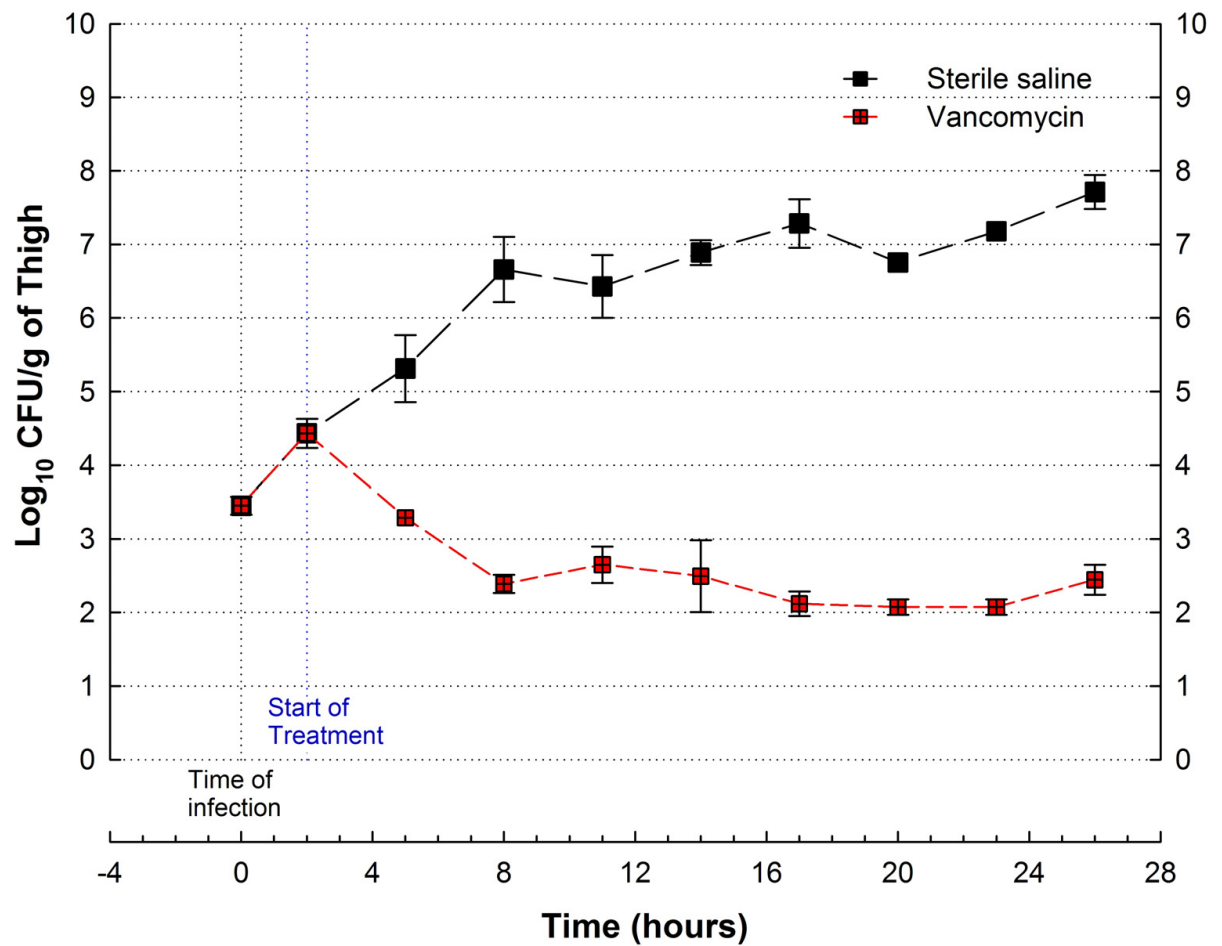
In vivo efficacy of vancomycin 1200 mg/kg/day (red squares) compared with sterile saline (black squares) against *S. aureus *GRP-0057 in the neutropenic mouse thigh infection model. Immunosupression achieved with a low-dose (250 mg/kg) cyclophosphamide regimen; treatment administrated every 3 hours by subcutaneous injections. Each data-point represents the mean ± standard deviation from both thighs of 2–4 mice (unseen error bars are contained within symbols).

**Figure 3:**
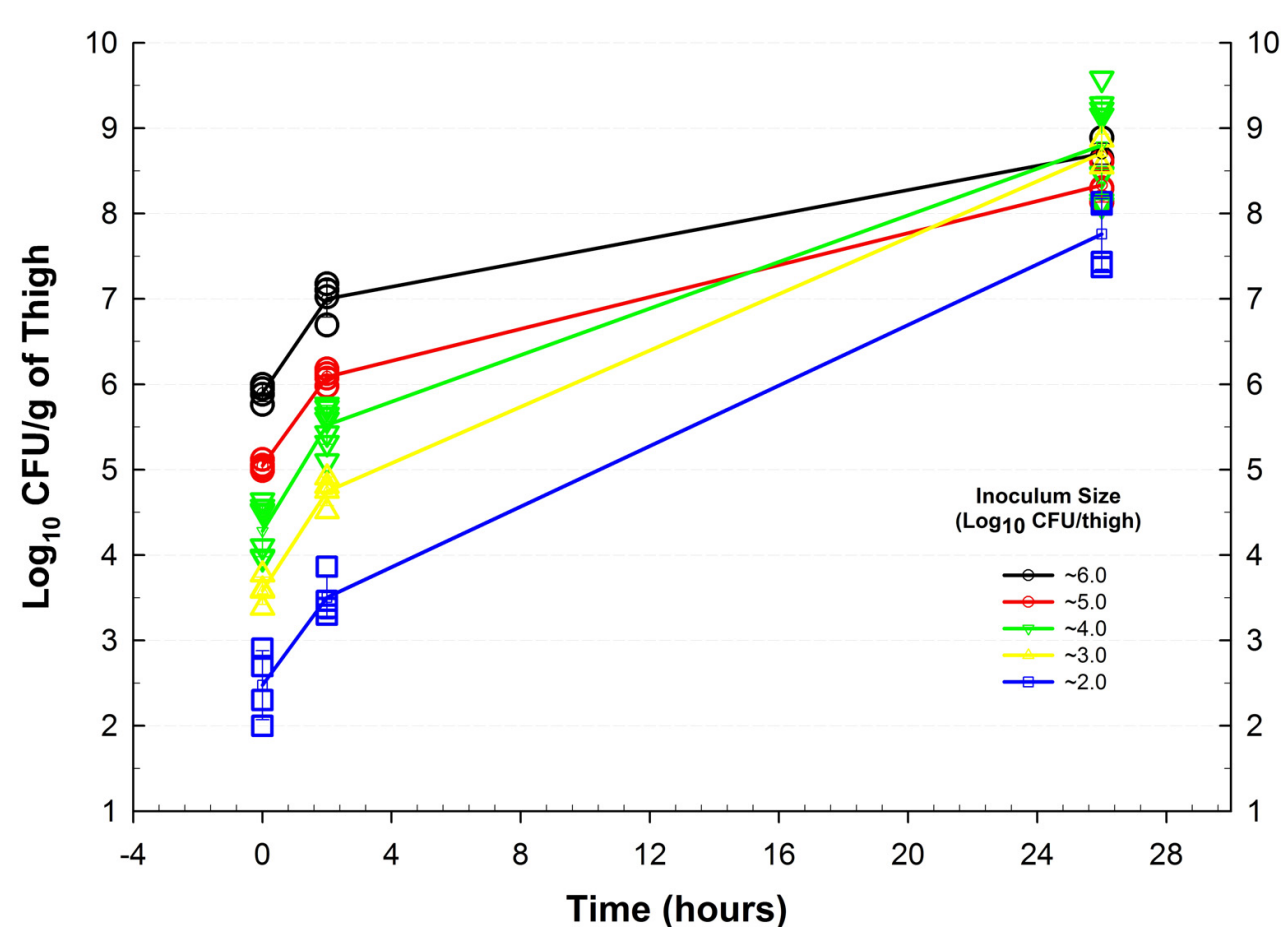
Impact of the inoculum size in bacterial growth using the neutropenic mouse thigh infection model with *E. coli *SIG-1. A low-dose cyclophosphamide regimen allowed successful induction of systemic infection inoculating as little as 100 microorganisms per thigh. Each data-point represents one thigh (2–4 mice per time-point), with the growth curve passing through the mean ± standard deviation.

**Figure 4:**
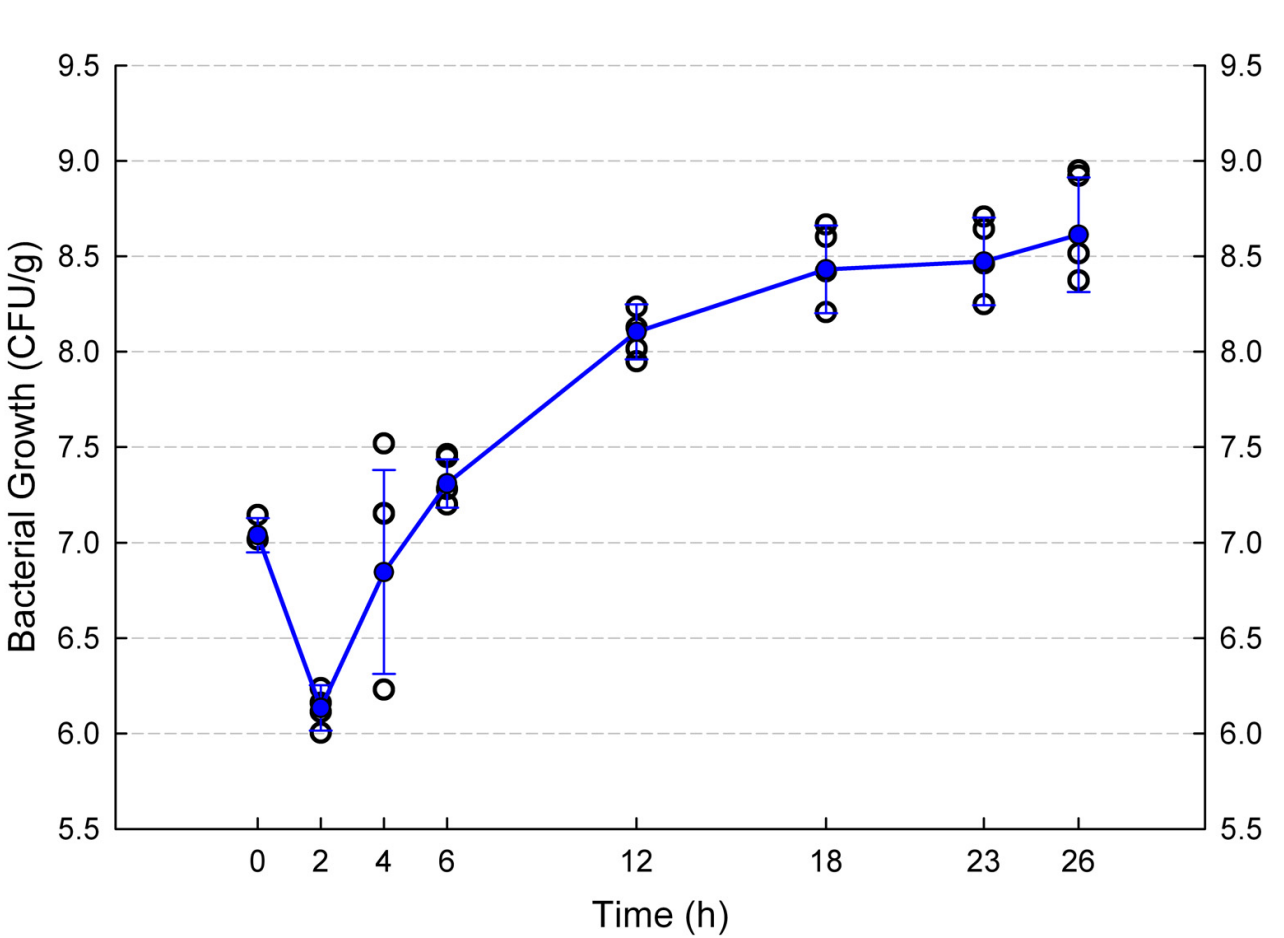
Growth curve of *B. fragilis *ATCC 25285 in the thighs of mice rendered neutropenic under a low-dose cyclophosphamide protocol. This model took advantage of bacterial synergism by inoculating the animals with a mixed culture (+ *E. coli *SIG-1; not shown in this graph). Empty black circles represent one thigh (2 mice per time-point); filled blue circles and lines are means ± standard deviations.

Figure 2 shows in vivo growth curves of *S. aureus *GRP-0057 in neutropenic mice, and response to treatment with vancomycin under the thigh infection model. Bacteria displayed a constant rate of growth with minimal variance that provides reliable data for antibiotic efficacy with this model, allowing the use of only two animals per dose and reducing untreated controls to the time of starting (2 mice) and ending (2 mice) therapy. Neutropenia also allowed for reproducible induction of infection independently of the inoculum size, as demonstrated by the experiments with *E. coli *SIG-1 (Figure 3). The model of anaerobic synergistic infection was also successful and reproducible, even with the desirable intra and inter-individual variation characteristic of outbred mice (Figure 4). Lethality for these models approximates 100% 48 hours after infection (data not shown).

**Figure 5:**
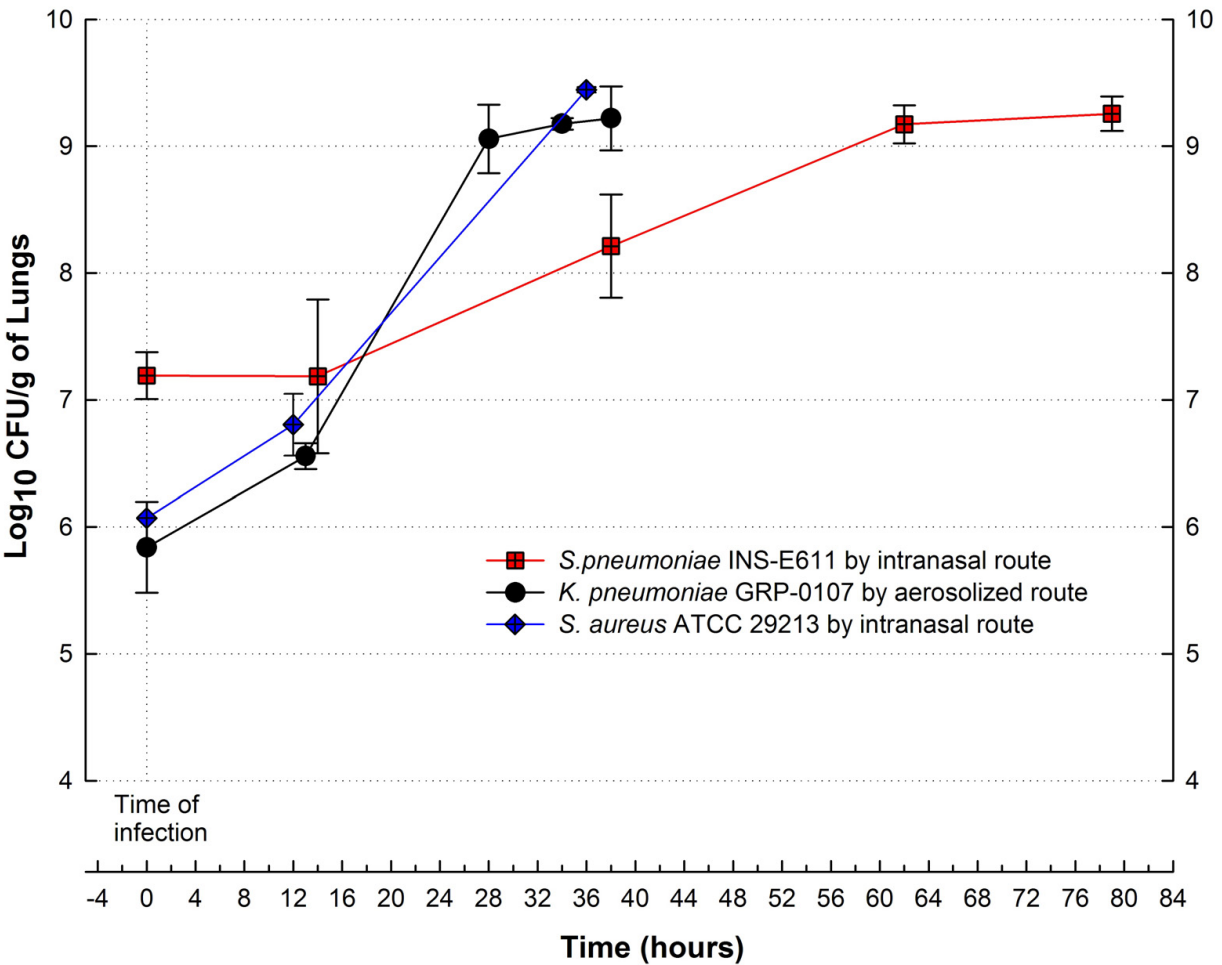
Comparison of bacterial growth of three human pathogens in the lungs of neutropenic mice inoculated by nasal instillation of *S. aureus *ATCC 29213 and *S. pneumoniae *INS-E611 or aerosolized *K. pneumoniae *GRP-0107. The apparent difference in the extension of the growth curves is due to the fact that mice infected with pneumococci survived the model, while the other organisms killed the animals 24–48 hours after infection; other details for each model in the text.

Lung infection models are difficult to reproduce, particularly with penicillin-resistant *S. pneumoniae *(PRSP). However, we found that neutropenic mice develop progressive pneumonia with all pathogens used (Figure 5) without recurring to nonphysiologic or traumatic methods for inoculation. Pneumonia was uniformly lethal 72 hours after infection with *K. pneumoniae *and *S. aureus*, but most PRSP infected mice had recovered their health by day 11, when neutrophil values had returned to normal (data not shown).

**Figure 6:**
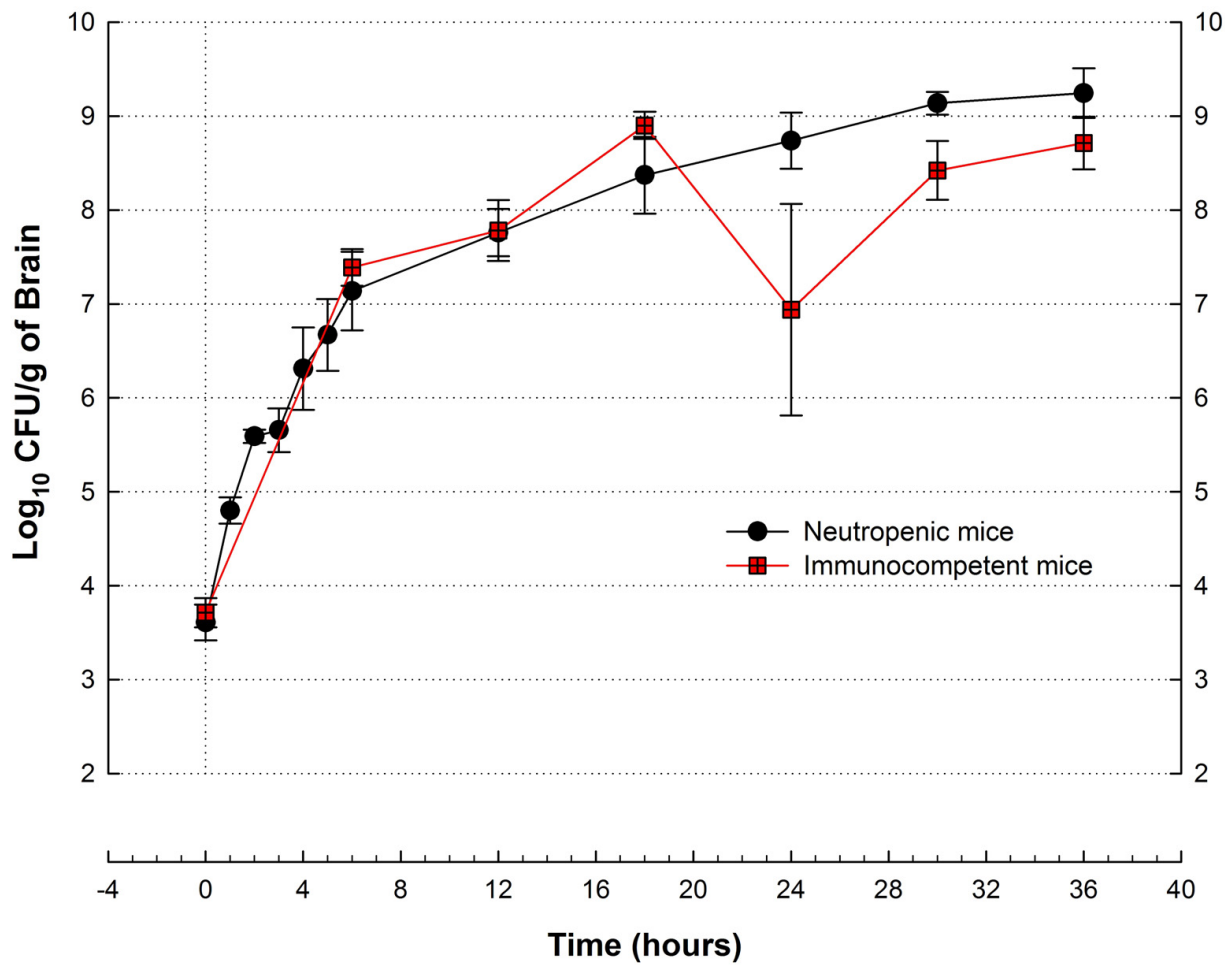
Influence of severe neutropenia (<10 cells/mm^3^) in the growth curves variance of *S. pneumoniae *GRP-0056 in a murine model of acute meningoencephalitis. Besides the brain (shown here), variance in mice survival and bacterial numbers in other organs (cerebellum, cerebrospinal fluid) is also smaller for neutropenic mice (black circles) compared with immunocompetent animals (red squares). Data-points represent means ± standard deviations from 2–10 mice.

Meningoencephalitis was easily induced to immunocompetent mice, allowing us to compare bacterial growth variance in the brain of animals treated with the low-dose CPM regimen against that of normal mice (Figure 6). Fitted to a polynomial cubic equation, the growth curves of *S. pneumoniae *GRP-0056 in the brains of neutropenic and normal mice favored the first group with smaller variation (standard error of the estimate, 0.3522 and 0.8194, respectively) and greater correlation between dependent (growth) and independent (time) variables (adjusted R^2^, 0.9618 and 0.7876, respectively).

## Discussion

The results of this study provide the first comprehensive characterization of the effects of a commonly used, low-dose CPM regimen on peripheral blood leukocytes in female outbred mice. It allowed successful and reproducible induction of diverse models of infectious diseases, independently of the method of inoculation, the pathogen used, or its susceptibility pattern.

CPM, a chemotherapeutic agent, has several effects that can impair host resistance, but the decrease in the number of circulating granulocytes is probably one of the most important factors for its use in different animal models, because it promotes severe infection (e.g. sepsis) and allows close quantification of the intrinsic efficacy of antibiotics. Previous research on animal infection models using irradiated mice showed that bacterial growth is inversely related to the number of granulocytes in peripheral blood [21,22], and other authors demonstrated with the thigh infection model that there was a significant difference in CFU numbers at the site of infection between mice treated with CPM (250 mg/kg) and non-neutropenic mice [23].

Previous works have also shown that, after profound neutropenia, mice recovered from CPM immunosupression with an increment in the number of blood leukocytes [24-26]. We observed a similar phenomenon at day 11 when all mice, previously neutropenic, displayed relative neutrophilia compared with baseline values (Table 1 and Figure 1). It is important to bear this fact in mind when designing or modifying the duration of the experimental model, particularly when using survival as the end-point. The low-dose CPM regimen is best suited for animal models equal to or shorter than 4 days after infection, independent of the selected endpoint (number of remaining microorganisms or animal survival). Models longer than 5 days would have both of these endpoints affected by the incipient recovery of the immune system. If such a model was necessary, a third dose of CPM might be required.

Severe neutropenia is not necessarily defined by the same value in humans and mice. The lower limit of neutrophil count in the normal mouse is 110 cells/mm^3 ^[20], while for normal humans it is 1800 cells/mm^3 ^[27]. Although 100 neutrophils (5.56% of 1800) imply severe neutropenia for a patient, the same value is still very close to normal for the mouse (90.9% of 110). Based on this comparison, we preferred to use 10 neutrophils/mm^3 ^(9.1% of 110) to define severe neutropenia for the mouse.

We found that the first cell line affected by the low-dose CPM regimen was the granulocytic followed by the lymphocytic line. While neutropenia is obtained with the first dose (150 mg/kg), the second dose (100 mg/kg) must be administered to have lymphopenic mice. On the other hand, monocyte counts were reduced 96% by day 5 with our low-dose intraperitoneal CPM regimen, but the absolute cell number did not reach the inferior normal limit for the mouse (10 cells/mm^3^). This is not surprising, since a previous work already showed that monocytopenia was induced only when the drug was administered by the subcutaneous route [7].

The use of various animals as models for microbiological infections has been a fundamental part of infectious disease research for more than a century. The data from animal models provide means for screening potential treatments and eliminating undesirable ones, narrowing the successful candidates for use in human studies. Due to the ease of handling and reproducibility, the neutropenic murine thigh infection model has been extensively used, especially to evaluate the relationship between host, drug and microorganism in the absence of immunity, which often acts as a confounding variable [16,28-30]. While inbred and outbred stocks have been employed for this model, the latter are preferred in pharmacology and toxicology studies because they represent better the genetic variability observed in human populations. Although there is a brief description of the neutropenia process after injecting low-dose CPM to inbred mice [14,31], the response of outbred mice had not been thoroughly described, and some authors feel more comfortable using the high-dose regimen [8-10]. The high-dose regimen requires greater CPM concentration per injection (250 mg/kg) or more than two injections, conditions implying unnecessary exposure of researchers and environment to this expensive carcinogenic agent [32,33], as well as greater risk of deadly peritoneal trauma and hemorrhagic cystitis for the animals [34].

Our results demonstrate that a low-dose CPM regimen induces severe neutropenia (≤10 cell/mm^3^) in outbred mice, sustained enough to reliably execute animal models of infection with different endpoints up to 4 days long. Two doses simplify drug administration, and minimize the aforementioned problems associated with high-dose CPM, without compromising the model performance.

## Conclusion

Previous results from a variety of papers suggest that intraperitoneal injections of CPM, administered 4 days (150) and 1 day (100 mg/kg) before experimental infection, induce neutropenia (≤100 neutrophils/mm^3^) in inbred mice and rats. Here, we showed that this low-dose regimen also induces profound and sustained neutropenia (<10 neutrophils/mm^3^) in outbred mice lasting at least 3 days. The use of a lower dose of CPM divided in two intraperitoneal injections is simpler, safer, and very effective to attain reproducible animal models of infection.

## Competing interests

The author(s) declare that they have no competing interests.

## Authors' contributions

OV conceived the study, directed its design and execution, obtained funding, reviewed, and re-wrote the final version of the manuscript. AFZ contributed in the study design, performed the analysis and interpretation of data, and prepared the manuscript with CAR. BES carried out the hematological analysis. CAR, MA and AXZ took care of the animals and carried out the experiments with assistance from the other authors.

## Pre-publication history

The pre-publication history for this paper can be accessed here:


